# Nature Versus Nurture: Does Proteostasis Imbalance Underlie the Genetic, Environmental, and Age-Related Risk Factors for Alzheimer’s Disease?

**DOI:** 10.3390/healthcare5030046

**Published:** 2017-08-22

**Authors:** Elise A. Kikis

**Affiliations:** Biology Department, The University of the South, Sewanee, TN 37383, USA; eakikis@sewanee.edu; Tel.: +1-931-598-1747

**Keywords:** Alzheimer’s disease, amyloid beta, ApoE, air pollution, particulate matter

## Abstract

Aging is a risk factor for a number of “age-related diseases”, including Alzheimer’s disease (AD). AD affects more than a third of all people over the age of 85, and is the leading cause of dementia worldwide. Symptoms include forgetfulness, memory loss, and cognitive decline, ultimately resulting in the need for full-time care. While there is no cure for AD, pharmacological approaches to alleviate symptoms and target underlying causes of the disease have been developed, albeit with limited success. This review presents the age-related, genetic, and environmental risk factors for AD and proposes a hypothesis for the mechanistic link between genetics and the environment. In short, much is known about the genetics of early-onset familial AD (EO-FAD) and the central role played by the Aβ peptide and protein misfolding, but late-onset AD (LOAD) is not thought to have direct genetic causes. Nonetheless, genetic risk factors such as isoforms of the protein ApoE have been identified. Additional findings suggest that air pollution caused by the combustion of fossil fuels may be an important environmental risk factor for AD. A hypothesis suggesting that poor air quality might act by disrupting protein folding homeostasis (proteostasis) is presented.

## 1. Alzheimer’s Disease and Public Health

Jean Calment of Arles, France lived to be 122 years old, making hers the longest confirmed lifespan of any human being. Her longevity can be attributed in part to an active lifestyle (despite smoking) and in part to genetics. As a testament to what is referred to as healthy aging—the Holy Grail of geriatric medicine and aging research—Calment lived on her own until the age of 110. Healthy aging involves maximizing lifespan while minimizing age-related medical conditions, and is thereby correlated with a high quality of life. It is notoriously difficult to achieve because aging in and of itself is a significant risk factor for a variety of what are aptly termed “age-related diseases” including diabetes, heart disease, cancer, and a number of neurodegenerative diseases including Alzheimer’s disease (AD) [1].

AD is the worldwide leading cause of dementia in the elderly, affecting nearly 50 million people according to Alzheimer’s Disease International [2]. The disease was first described in the early 1900s by Alois Alzheimer, who observed proteinaceous deposits and significant neurodegeneration in the postmortem brain of a 51 year-old patient. The patient originally presented with early onset dementia and suffered rapid cognitive decline over a period of a few years before her death [3]. Studies have shown that the memory center of the brain known as the hippocampus is one of the first areas affected by AD, such that the earliest disease symptoms involve forgetfulness. As the disease progresses and more brain regions become affected, memory loss turns more severe and reasoning, behavior, and emotional stability start to decline. Most AD patients have such difficultly with activities of daily living (ADL) that they are unable to live on their own and require either home care or residence in an assisted living facility [4].

Conventional wisdom reasonably presumes that the extent of ADL impairment reflects disease-severity. However, a recent study of 1029 patients at the Baylor College of Medicine found that while ADL impairment is strongly correlated with the risk of death for AD patients, it is often independent of other known biomarkers for AD severity. The authors of that study thereby suggest that treatments leading to an ADL improvement should be pursued, in some cases over those that treat other aspects of the disease [5].

Despite more than 100 years of research on AD, there is still no cure. Current medical interventions include disease-modifying drugs (DMDs) and less expensive drugs that are meant only to treat symptoms [6]. Many symptom-treating drugs result in mild cognitive benefits [7], while others target psychiatric and behavioral aspects of AD [8]. Ultimately, treatment regimens strive to maximize both the quality of life and the lifespan of patients. Determining quality of life for patients with severe dementia is a notoriously difficult task that usually involves interviewing both the patients and their caregivers [9]. In fact, caregivers with close relationships or significant interactions with AD patients may be in a better position than the patients themselves to provide feedback to doctors regarding the effects of various treatments.

Without a cure on the horizon, the number of people with AD or other age-related dementias will increase substantially over the next 20 years. This is partly because the baby boomer generation is just starting to reach retirement age. Furthermore, the medical infrastructure continues to improve, and mortality by infectious diseases is in decline. As the population ages, the next generation will be saddled with an unprecedented caregiving burden.

In addition to impacting earning potential and the ability to save for their own retirement, family members of AD patients serving as caregivers also suffer their own array of comorbidities, resulting in a declining health-related quality of life and an increasing number of emergency room visits as compared to non-caregivers [10]. Caregivers have also been shown to suffer from stress and psychiatric conditions, contributing to impaired functioning with respect to their own ADL [11].

The only way to alleviate this growing public health crisis is to find a cure, or at least better treatment options, for AD. In 2010, the only pharmaceuticals approved for the treatment of AD symptoms were cholinesterase inhibitors that increase acetylcholine levels and *N*-methyl-d-aspartic acid antagonists that inhibit glutamate signaling in the brain [4]. Much recent research has focused on elucidating the molecular mechanisms underlying AD, which has led to new treatment regimens as described below.

## 2. Genetic Risk Factors

### 2.1. Early-Onset AD

Studies aimed at elucidating the cell biological and biochemical mechanisms that underlie AD have revealed interactions between a number of genes, processes, and even cell types. Consequently, two AD patients may present with very similar symptoms but have different genetic predispositions. This suggests that there are multiple ways to arrive at the same endpoint, and thus no single explanation for the cause of AD. This naturally adds a layer of complexity to any proposed treatment regimens. Fortunately, there seem to be a few subtypes of AD with related underlying causes.

For example, a small subset—accounting for between one and five percent of AD cases—is caused by strikingly similar gene mutations leading to very aggressive early onset of symptoms (as early as 30 years of age) [12]. Such cases are aptly referred to as early-onset familial AD (EO-FAD). Studies in the 1990s revealed that the misfolding and aggregation of the amyloid-β (Aβ) peptide is a defining component of EO-FAD etiology.

The Aβ peptide is an abnormal cleavage product of the amyloid precursor protein (APP), whose normal cellular function is an area of active investigation [13]. Normally, APP is sequentially cleaved by the proteases α-secretase and γ-secretase at the cell surface, yielding normal non-toxic protein fragments referred to as p3 peptides. However, certain EO-FAD-associated genetic variants of APP are internalized into vesicles containing β-secretase (BACE1) and γ-secretase that together give rise to disease-associated Aβ peptides that are longer than p3 but derived from the same precursor. The inherently aggregation-prone Aβ peptides form amyloid fibrils and extracellular plaques in a concentration-dependent manner [14,15,16].

Not all Aβ peptides are the same length. In fact, the ratio of Aβ_1-40_: Aβ_1-42_ may be an important predictor of AD. Thus, other mutations that promote the production of Aβ_1-42_ are also associated with EO-FAD. Specifically, mutations in presenilin 1 and presenilin 2 alter the proteolytic site of γ-secretase causing it to produce more Aβ_1-42_, which is highly amyloidogenic [17].

Taken together, these findings led to the Aβ peptide becoming an important area of investigation and an important drug target for the treatment of AD. Aβ accumulation is associated with neuronal apoptosis, oxidative damage, synapse loss, and even seizures [18]. Nonetheless, drugs that specifically target the production of Aβ (e.g., by altering the activities of β-secretase or γ-secretase) have been strikingly ineffective as AD treatments. Even when drugs have succeeded at partially reducing Aβ levels in the brain, they did little to mitigate the cognitive effects of AD [19]. The next generation of pharmaceuticals includes immunotherapies that aim to enhance the clearance of the Aβ peptide. While such treatments have experienced early successes in clinical trials insofar as triggering a marked increase in anti-Aβ IgG levels, little concomitant cognitive improvement has been observed [20].

### 2.2. Late-Onset AD

Late-onset forms of AD (LOAD) are much more common than EO-FAD, accounting for approximately 95% of all instances of AD. Symptoms appear after the age of 65, with progressive deterioration of brain function, and death within and average of 10 years. LOAD is considered to be sporadic because it has no known genetic causes. However, recent efforts to understand the disease have uncovered a number of genetic risk factors.

The strongest and most consistent risk factor for LOAD is the presence of the ε4 allele of the apolipoprotein gene (*APOE*) such that carriers of the ε4 allele are at a nearly 12-fold increased risk of the disease compared to the general population [21]. There are three common *APOE* alleles in people, referred to as ε2, ε3, and ε4, which differ at two amino acid positions. The most common is the ε3 allele, which can thus be considered the reference allele. Carriers of the ε2 allele are at a reduced risk of LOAD, relative to those who are homozygous for the ε3 allele [22].

The normal function of the 299-amino-acid, 34kD, ApoE protein is to regulate the transport of lipids between cell types. In the brain, ApoE is produced by astrocytes and acts as a transporter of cholesterol to neurons by binding to ApoE receptors [23]. Recent findings suggest that the various *APOE* alleles may have both Aβ-dependent and Aβ-independent mechanisms by which they contribute to LOAD.

### 2.3. Aβ-Dependent Pathways for ApoE

Much evidence points to ApoE modulating the levels of Aβ in the brain. In fact, even some asymptomatic homozygous carriers of the *APOE* ε4 allele have been shown to accumulate more Aβ fibrils than individuals with other genotypes [24,25]. Furthermore, ApoE was first identified based on its ability to physically interact with Aβ. Mechanistically, it seems that the ApoE protein is able to trigger the degradation of Aβ in mouse models of AD in a manner dependent on its isoform and lipidation state [26]. 

If ApoE acts through Aβ to increase the risk of AD in an allele-dependent manner, then should an additive effect not be observed in patients carrying EO-FAD mutations? Consistent with this, a recent study of patients with mutations in presenilin 1 revealed that the age of onset of disease symptoms was delayed in patients carrying the protective *APOE* ε2 allele [27]. At face value, these findings are consistent with the “amyloid cascade hypothesis” that posits that all forms of AD are caused by the toxic effects of insoluble Aβ deposits in the brain. However, recent findings point to ApoE contributing to AD in other ways as well.

### 2.4. Aβ-Independent Pathways for ApoE

Synapses are inherently unstable, with relatively short half-lives in healthy adult brains. This turnover is needed to maintain the plasticity of neuronal connections, which is especially important for learning and memory–functions of the brain that are disrupted in AD patients. Astrocytes are phagocytic cells that play a major role in mediating synapse turnover [28]. Moreover, astrocytes secrete ApoE [29]. Recent evidence suggests that the *APOE* ε4 allele impairs the ability of astrocytes to prune neurons [28]. Furthermore, mice expressing the human *APOE* ε4 allele are reported to have a significantly higher number of senescent synapses than *APOE* ε3 mice [28]. Importantly, while astrocytes have been shown to phagocytose the Aβ peptide [30], synapse pruning does not directly involve Aβ.

Another possible Aβ-independent role for *APOE* ε4 involves phospholipid metabolism. Dysregulation of lipid metabolism is known to correlate with AD [31,32]. However, the mechanism was not understood until recently, nor was it clear that it was a cause of disease rather than an epiphenomenon. A recent report demonstrated that certain phosphoinositols accumulate to abnormally high levels due to the upregulation of phospholipid degrading enzymes in mice expressing the human *APOE* ε4 allele [33]. As evidence that this upregulation is causally linked to disease, the authors showed that downregulating this enzyme rescued the *APOE* ε4-associated cognitive defects in mice [33].

Since LOAD can manifest in both Aβ-dependent and Aβ-independent manners, it should not be surprising that treatments that exclusively target Aβ levels have not always been effective [34]. Instead, effective treatments will likely depend on patients’ individual brain chemistries.

## 3. Environmental Risk Factors

At least half of all AD patients do not have the previously identified disease-associated mutations in APP, presenilin 1, presenilin 2, or ApoE. Therefore, there must be additional explanations for what causes AD and what can be considered a risk factor. Could the environment be a risk factor? Do certain environmental conditions exacerbate the contribution of genotype to AD risk? The last few years have witnessed a rise in the number of studies that link air pollution to AD and related dementias. With the President of the United States recently announcing his intention to pull out of the Paris Climate Accord, the question of air quality as a risk factor for AD is especially timely.

An epidemiological study was performed on people of European descent living in urban areas in the continental United States. Subjects were 65–75 years old and had no signs of dementia at the time the study began. Cognitive impairment along with the extent of exposure to small particulate matter 2.5 μm or less in diameter (PM_2.5_)—produced by burning fossil fuels—was documented over time. Women living in areas with high PM_2.5_ in the air were shown to be at an increased risk for dementia. This risk was significantly higher in those individuals carrying two copies of the *APOE* ε4 allele [35]. A similar study of people living near major roads in Canada pointed to poor air quality being a risk factor for certain types of age-related dementia. Interestingly, PM_2.5_ did not seem to be a risk factor for Parkinson’s disease (PD), meaning that it is not a general risk factor for all types of neurodegenerative disorders [36]. Instead, PM_2.5_ may trigger cognitive decline by acting on specific proteins, such as Aβ. 

While Aβ aggregation was not directly monitored in either of the epidemiological studies described above, further studies with AD mice showed that PM_2.5_ exposure triggers Aβ aggregation in a manner dependent on the *APOE* genotype [35]. Together, these data point to a disease mechanism by which *APOE* ε4 and poor air quality work together to cause AD in an Aβ-dependent manner.

Another recent mouse study involved exposure to a combination of PM_2.5_ and SO_2_ [37], which are known to co-exist as products of fossil fuel combustion [38]. The authors reported an increase in tau hyperphosphorylation leading to the accumulation of neurofibrillary tangles—phenomena associated with several neurodegenerative diseases, including AD and frontotemporal dementia. As such, the mechanism by which air pollution acts as a risk factor for AD may be multifaceted and may differ depending on the molecular components contaminating the air.

The above evidence linking air quality to AD is tantalizing; however, the mechanism(s) underlying the phenomenon is unknown. Furthermore, while the majority of recent findings are centered on AD, insufficient data exist to ascertain the extent to which other neurodegenerative diseases may likewise be exacerbated by PM_2.5_ in the air.

With respect to the mechanism of action, it seems likely that small particulate matter produced by the combustion of fossil fuels may be an important source of proteotoxic stress that contributes to an imbalance in protein folding homeostasis (proteostasis). This hypothesis is based on the understanding that AD is a protein folding disorder with Aβ peptide misfolding at its core [39]. Nonetheless, PM_2.5_ has never been tested for its propensity to trigger protein misfolding, either generally or of Aβ in particular, and will be an important new area of investigation.

## 4. Conclusions

AD is the worldwide leading cause of dementia and a major age-related disease. As memory loss and cognitive decline are hallmarks of AD, persons living with the disease experience significant ADL impairment, resulting in the need for full-time care. Thus, AD impacts both aging patients and caregivers. Since the early 1990s, a major aim in AD research has been to identify genetic causes or genetic risk factors, with the hope that such knowledge would pave the way for the development of pharmaceuticals that target the underlying mechanism(s) of disease. Over the last 25 years, much progress was made toward understanding the genetics. Namely, three genes in which mutations cause EO-AD have been described, leading to the Aβ peptide being launched to the position of highest priority with respect to possible disease mechanisms. Additionally, the strongest genetic risk factor for sporadic LOAD is now thought to be *APOE* ε4. While the *APOE* genotype is certainly not predictive of the disease, the mechanism by which it increases the risk of disease is an area of active investigation. Recent data suggest that particulate matter generated by the burning of fossil fuels interacts with the *APOE* genotype by exacerbating its known Aβ-dependent functions, leading to an increase in Aβ-aggregation and consequently to an increase in neurodegeneration. This may be caused by an imbalance in proteostasis and protein quality control.
